# Formulation process, physical stability and herbicidal activities of *Cymbopogon nardus* essential oil-based nanoemulsion

**DOI:** 10.1038/s41598-022-14591-2

**Published:** 2022-06-18

**Authors:** Naphat Somala, Chamroon Laosinwattana, Montinee Teerarak

**Affiliations:** Department of Plant Production Technology, Faculty of Agricultural Technology, King Mongkut’s Institute of Technology Ladkrabang, Bangkok, 10520 Thailand

**Keywords:** Chemical biology, Plant sciences, Nanoscience and technology

## Abstract

Essential oil-based bioherbicides are a promising avenue for the development of eco-friendly pesticides. This study formulated nanoemulsions containing citronella (*Cymbopogon nardus*) essential oil (CEO) as an herbicidal product using a high-pressure homogenization method with hydrophilic-lipophilic balance (HLB) values ranging 9–14.9 for the surfactant mixture (Tween 60 and Span 60). The CEO was high in monoterpene compounds (36.333% geraniol, 17.881% *trans*-citral, 15.276% *cis*-citral, 8.991% citronellal, and 4.991% *β*-citronellol). The nanoemulsion at HLB 14 was selected as optimal due to having the smallest particle size (79 nm, PI 0.286), confirmed by transmission electron microscopy. After 28 days of storage, particle size in the selected formulation changed to 58 and 140 nm under 4 °C and 25 °C, respectively. Germination and seedling growth assays with *Echinochloa crus-galli* showed that the nanoemulsion exerted a significant dose-dependent inhibitory effect at all tested HLBs (9–14.9) and concentrations (100–800 µL/L). The inhibitory effect was greatest at HLB 14. Treatment of *E. cruss-galli* seed with the HLB 14 nanoemulsion significantly reduced seed imbibition and *α*-amylase activity. Our findings support that CEO nanoemulsions have a phytotoxic effect and hence herbicidal properties for controlling *E. cruss-galli*. Accordingly, this nanoemulsion may have potential as a bioherbicide resource.

## Introduction

Exploring and developing new bio-based herbicides to replace synthetic herbicides is necessary for maintaining sustainable agriculture, protecting the environment, and decreasing pollution. Additionally, synthetic herbicides have only one or a few modes of action. Hence the continued of synthetic herbicides on weeds can contribute to the development of resistance to chemical herbicides^1^. Essential oils (EOs) are lipophilic natural substances containing secondary metabolites, i.e., monoterpene alcohols or oxygenated monoterpenes, which are obtained from plants and frequently reported as having herbicidal activities related to their allelochemical compounds^1–3^. These natural materials are good candidates as a source of potential biocontrol products^4–8^. Regarding herbicidal effects in particular, EOs have variously been reported to cause oxidative damage, ion leakage, decreased cellular respiration, waxy cuticular layer removal, and mitosis inhibition^9,10^. Herbicides based on plant EOs have been demonstrated effective against a wide range of weeds, and have promise as natural alternatives to nonselective herbicides^11^.

The essential oil of citronella (*Cymbopogon nardus*) (CEO), a perennial aromatic grass, has been widely studied as a bioherbicide product^10,12,13^. Citronella oil has been demonstrated effective against seed germination in weeds like billy goat weed (*Ageratum conyzoides*), coffee weed (*Cassia occidentalis*), and parthenium ragweed (*Parthenium hysterophorus*)^14^. Suwitchayanon, et al.^15^ also reported that *C. nardus* leaves showed inhibitory effects on growth of common weeds in agriculture fields, namely Italian ryegrass (*Lolium moltiflorum* Lam.) and jungle rice (*Echinochloa colonum* L.). Therefore, CEO has the potential to be used as a natural herbicide.

Historically, EO-based products have been formulated into emulsions for application as herbicides. Since EOs are composed of small amphiphilic molecules, they are able to cross the cell wall mesh and directly interact with the plant plasma membrane (PPM). Recently, a myriad of researchers has been utilizing nanotechnology and various emulsification preparation methods to formulate natural herbicides containing EOs with reduced emulsion particle size^1,16^. The benefit of nanoemulsions is their long kinetic stability compared to macroemulsion which is weakly kinetically stable^17^. Nanoemulsions can be produced using either low- or high-energy emulsification methods and with various emulsifier agents such as surfactants^18^ or biopolymers^19^. The characteristic properties of nanoemulsions (i.e., droplet size, PI, and zeta potential) are influenced by factors such as the type and ratio of EO and surfactant, the hydrophilic-lipophilic balance (HLB) of the EO, and preparation method^20^. Importantly, the nanoemulsion with the smallest droplet size, which is the most stable, is obtained when the HLB value of the surfactant mixture (Smix) is near that of the EO.

While CEO has been reported to show herbicidal potential against weeds^10,21^, to the best of our knowledge, no one has yet investigated the herbicidal activity of a nanoemulsion formulation comprised of CEO with a nonionic Smix at optimal HLB. Hence, this present work aimed to optimize the Smix (Tween 60 and Span 60) HLB and concentration so as to formulate a CEO nanoemulsion with tiny droplet diameter, long kinetic stability, and improved herbicidal potential. We further investigated the herbicidal activity of the formulated nanoemulsion against *E. cruss-galli*, along with some of its physiological effects.

## Material and methods

### Essential oils and chemical materials

Citronella (*Cymbopogon nardus*) essential oil was purchased from Thai—China Flavours and Fragrances Industry Co., Ltd. (Bangkok, Thailand), CAS No. : 8000-29-1. The emulsifier agents Tween 60 and Span 60 were purchased from Chemipan Corporation Co., Ltd. (Bangkok, Thailand). Deionized water was obtained from RCI Labscan (Ireland) and used in all experiments.

### Identification of essential oil constituents by gas chromatography/mass spectrometry (GC/MS)

CEO components were identified using gas chromatography in conjunction with mass spectrometry (GC–MS). In detail, the EO was diluted in ethyl acetate and analyzed by means of an Agilent 6890 N gas chromatograph having an Agilent 5973 mass detector equipped with an HP-5 silica capillary column (30 m × 0.25 mm ID, 0.25 µm film thickness). The oven temperature program consisted of an initial 40 °C for 3 min followed by an increase with heating rate 10 °C/min to 100 °C, then a further increase at 5 °C/min to 260 °C, which was held for 5 min. Helium was used as the carrier gas at a flow rate of 1 mL/min. MS analysis was carried out over a detection range of 30–500 amu. The sample (0.2 µm) was injected with a split ratio of 50:1. The injector and detector were maintained at respective temperatures of 250 °C and 270 °C. Individual compounds were identified by MS and their identity confirmed by comparison of Kovat’s retention index with reference to a homologous series of n-alkanes. Percentage composition was determined based on GC peak area and retention time as calculated by a Shimadzu CR6A data processor.

### Preparation of nanoemulsion formulations

CEO was formulated into oil-in-water (O/W) nanoemulsions using a high-energy emulsification method at room temperature (25 ± 2 °C) with a surfactant mixture (Smix) as the emulsifier agent, namely polyethylene glycol sorbitan monostearate (Tween 60; HLB = 14.9) as surfactant and sorbitan monostearate (Span 60; HLB = 4.7) as co-surfactant. The nanoemulsion consisted of a dispersed phase (2%w/v of CEO and 2%w/v of Smix) and a continuous phase (96%w/v of water). In producing the dispersed phase, Span 60 and CEO were first mixed with a magnetic stirrer at 1500 rpm for 20 min. Tween 60 was added into the mixture and stirred continuously for 3 min. Then, water was added and the solution stirred continuously for 10 min. The resulting coarse emulsion was prepared into a fine emulsion using an M-110P microfluidizer processor (Microfluidics, Newton, MA, USA) at 25 °C, 15,000 psi, 1 cycle to form uniform particles and reduce droplet size. The microfluidizer interaction chamber was a Z type with diameter of 87 µm. 200 mL of the coarse emulsion was fed to the microfluidizer processor. For a cycle, this device pumped the coarse emulsion towards an interaction chamber which is equipped with micro-channels. And the high-shear forces form the reduction of droplet size of the emulsion^22^. The obtained fine emulsion was stored at room temperature and evaluated in further experiments.

### Optimization of HLB value

To determine the required hydrophilic-lipophilic balance (rHLB) of citronella oil, Smix with predicted HLB values ranging 9–14 was prepared as stated above by mixing different ratios of Tween 60 and Span 60 (Table 1). Predicted HLB values were determined using Griffin’s formula^23^:

$$ {\text{HLB}}_{{{\text{Smix}}}} = {\text{ HLB}}_{{\text{A}}} \times {\text{ X}}_{{\text{A}}} + {\text{ HLB}}_{{\text{B}}} \times \left( {{1 }{-}{\text{ X}}_{{\text{A}}} } \right) $$

where X_A_ is the mass fraction of surfactant A.

**Table 1:** Tested ratios of Smix constituents Tween 60 and Span 60 and corresponding HLB.

HLB values of Smix	Tween 60 (%w/w)	Span 60 (%w/w)
9	42.2	57.8
10	52.0	48.0
11	61.8	38.2
12	71.6	28.4
13	81.4	18.6
14	91.2	8.8
14.9	100	–

Then, nanoemulsions were prepared with a constant CEO concentration of 2% w/v. The Smix concentration was likewise held at 2% w/v, and optimized later. Formulations were analyzed in terms of particle size and polydispersity index (PI) to determine the rHLB value of CEO. The selected formulation was stored at room temperature.

### Characterization of formulations

#### Droplet analysis

The formulations were evaluated in terms of particle size (Z-average) and PI by means of a dynamic light scattering (DLS) technique using a Nanoplus 3 (MICROMERITICS, Japan). Measurement conditions consisted of a fixed scattered angle of 165° and temperature at 25 °C. The obtained PI values were less than 0.5, indicating a droplet size distribution that is homogenous and adequate for agriculture^24^. The zeta potential, reflective of electrophoretic properties, was assayed to predict nanoemulsion stability due to electrostatic repulsion^4^. The nanoemulsion treatments were diluted (1:9) with DI water before use. Measurement conditions for zeta potential included a fixed scattered angle of 15° and temperature at 25 °C. Each measurement was computed in five replications using the program nanoPlus version 5.10/3.00.

#### Surface tension and pH measurement

The surface tension of the nanoemulsion was investigated by means of the Wilhelmy plate method using a DY-300 surface tension meter (Kyowa Interface Science, Japan) under a controlled temperature of 25 ± 1 °C. The pH was measured using a Consort C860 conductivimeter (Consort, Belgium). The observed pH value of a nanoemulsion follows from the determination of its stability due to alteration of pH in the presence of chemical reactions. The pH meter was calibrated using standard buffers of pH 4, 7, and 9. All measurements were collected in triplicate.

#### Rheological measurements

Rheological properties of CEO nanoemulsion at HLB 14 were determined using a MCR 302 Modular Compact Rheometer (Anton Paar) for the shear rate range of 0–250 s^−1^ in 600 s. 12.5 mL of the emulsion was performed in the rheometer.

#### Nanoemulsion stability and morphology

Kinetic stability was observed in the separation of layers or sedimentation by centrifuge. The nanoemulsion was centrifuged using an MPW-260R (MPW, Poland) at 5000 rpm for 15 min^18^, after which emulsion stability was evaluated based on droplet size, PI, and zeta potential measurement. The morphology of the nanoemulsion was determined using transmission electron microscopy (TEM) (HITACHI HT7700, Japan) operating at a voltage of 80 kV. A drop of nanoemulsion was allowed to settle on a carbon-coated grid for 10 min, then treated with a drop of 2% uranyl acetate and left for 30 s. The appearance of particle droplets was visualized using ImageJ software.

#### Storage study

The nanoemulsion was stored in a glass bottle at temperatures of 4, 25, and 45 °C for periods of 7, 14, 21, and 28 days; afterwards, the droplet size, PI, and zeta potential value were determined.

### Herbicidal activity of the nanoemulsion

#### Preparation of test seeds

Mature seeds of barnyard grass (*Echinochloa crus-galli* (L.) Beauv.) were collected from paddy fields at Ladkrabang, Bangkok, Thailand. All plant samples were identified by using comparative macro and micro-morphology using key characteristics in the Flora of Thailand and related documents. Also, they were compared with type specimens *E crus-galli* (holotype K000245284) at Kew Herbarium. The research on this plant species has comply with relevant institutional, national, and international guidelines and legislation.

The seeds were kept at room temperature for three months. To break dormancy, seeds were incubated in a hot-air oven at 45 °C for 48 h. Afterwards, the seeds were tested for their germination capability, which yielded a germination percentage of 95%.

#### Germination bioassays

The effect of the nanoemulsion on seed germination and seedling growth was evaluated by Petri dish bioassay under laboratory conditions. Nanoemulsion treatments were formulated with HLB numbers of 9-14.9 and CEO concentrations of 100, 200, 400, and 800 µL/L. Five milliliters of each treatment solution was added to an individual 9-cm-diameter Petri dish with double germination paper. Twenty seeds of *E. crus-galli* were placed in each dish and the dishes sealed with Parafilm. Then, the dishes were incubated for seven days in a growth chamber (LAC-1075-N, Longyue, Shanghai) at 27 ± 2 °C, 12/12 h light/dark, and humidity of about 80%. A solution of Smix in water served as a control. Germination count and root and shoot lengths (cm) were recorded after the seven days.

#### Seed imbibition

The optimal nanoemulsion was selected for evaluation of seed imbibition. Treatment formulations consisted of 0, 100, 200, 400, and 800 µL/L CEO. Water was used as a control. Seed imbibition was carried out according to Turk and Tawaha^25^ with modification. Briefly, 0.1 g (W_1_) of *E. crus-galli* seeds were soaked in the treatment solution for 24, 36, and 48 h. After incubation, the seeds were washed and weighed (W_2_). Seed imbibition percentage was then determined as follows:

$$ {\text{Seed imbibition }}\left( \% \right) \, = \, \left[ {\left( {{\text{W2 }} - {\text{ W1}}} \right)/{\text{W1}}} \right] \, \times { 1}00 $$

#### α-Amylase activity assay

Evaluations of α-amylase activity used the optimal nanoemulsion with 0, 100, 200, 400, and 800 µL/L CEO. Water was used as a control. *E. crus-galli* seeds were soaked in each treatment solution for 12, 24, and 36 h. Afterwards, the seeds were washed with distilled water, grained with 4 mL of 0.1 M CaCl_2_ in an ice bath (2–4 °C), and finally centrifuged at 10,000 rpm for 20 min at 4 °C. The supernatant was stored at 4 °C for the enzyme activity assay. *α*-Amylase activity was measured using the dinitrosalicylic acid (DNS) method as reported by Sadasivam and Manickam^26^. The reaction was started by mixing 1 mL of enzyme extract and 1 mL of 1% soluble starch in acetate buffer solution at pH 5.5. Then, the reaction solution mixture was incubated at 37 °C for 15 min. After incubation, the reaction was stopped by adding 1 mL of DNS reagent, which consisted of 40 mM 3,5 dinitrosalicylic acid, 0.4 N NaOH, and 1 M K-Na tartrate. The mixture was boiled at 100 °C for 5 min, then cooled in an ice bath. Finally, the absorption at 560 nm was measured using a UV/Vis spectrophotometer (Thermo Fisher Scientific, USA) and the *α*-amylase activity calculated and expressed as μmol maltose/min/g(FW).

### Statistical analysis

All experiments utilized a randomized complete block design in four replicates. Data are expressed as mean value ± standard deviation (SD). Means were compared using Tukey’s multiple range tests (*p* < 0.05).

## Results and discussion

### Determination of the chemical composition of CEO

CEO is a complex compound that has active ingredients appropriate for natural herbicide products. GC–MS analyses identified 28 components constituting 99.72% of the total CEO. The major constituents were of the monoterpene class, namely geraniol (36.333% of total volatiles), *trans*-citral (17.881%), *cis*-citral (15.276%), citronellal (8.991%), *β*-citronellol (4.991%), and citral diethyl acetal (4.603%) (Table 2). The chemical composition of CEO as determined here is in agreement with the prior report of Nakahara, et al.^27^, which found the major chemical constituents of essential oil from *C. nardus* to be geraniol (35.7%), *trans*-citral (22.7%), *cis*-citral (14.2%), geranyl acetate (9.7%), citronellal (5.8%), and citronellol (4.6%). Meanwhile, Timung, et al.^28^ reported the main compounds of the oil from leaves of Java citronella (*C. winterianus* Jowitt) as citronellal (55.24%), geraniol (26.29%), and citronellol (13.41%). However, the variation in these reported chemical compositions can be attributed to several influencing factors such as species, harvest stage, genetic differences, climatic and environmental conditions, and the extraction method.

**Table 2:** Chemical composition of EO from citronella leaves.

Number	Class	Constituents	RT^a^ min	%
1	Monoterpene	Camphene	6.144	0.15
2		Methyl heptenone	6.760	0.917
3		*β*-myrcene	6.83	0.048
4		Octanal	7.024	0.062
5		Limonene	7.488	0.135
6		2,6-Dimethyl-5-heptenal	7.876	0.128
7		4-Nonanone	8.179	0.987
8		(–)-Linalool	8.621	1.175
9		Citronellal	9.479	8.991
10		*trans*-Caran-4-one	9.905	1.11
11		Dacanal	10.24	0.406
12		*β*-Citronellol	10.607	4.991
13		*cis*-Citral	10.833	15.276
14		Geraniol	11.065	36.333
15		*trans*-Citral	11.27	17.881
16		Citronellyl acetate	12.312	0.371
17		Eugenol	12.441	0.276
18		Geranyl acetate	12.722	1.841
19		Neral dimethyl acetal	13.169	2.328
20		Caryophyllene	13.358	0.767
21		Citral diethyl acetal	13.45	4.603
22	Sesquiterpene	Humulene	13.795	0.113
23		(–)-Germacrene D	14.13	0.085
24		*α*-Cadinene	14.518	0.188
25		Cadinene	14.605	0.069
26		Elemol	14.923	0.081
27	Oxygenated sesquiterpene	Caryophyllene oxide	15.403	0.377
28		(–)-*α*-Cadinol	16.034	0.034
	Monoterpene			98.776
	Sesquiterpene			0.536
	Oxygenated sesquiterpene			0.411
	Total			99.72

### Optimization of HLB values

#### Effect of Smix on droplet size, PI, and zeta potential value of CEO nanoemulsion

**Figure 1:**
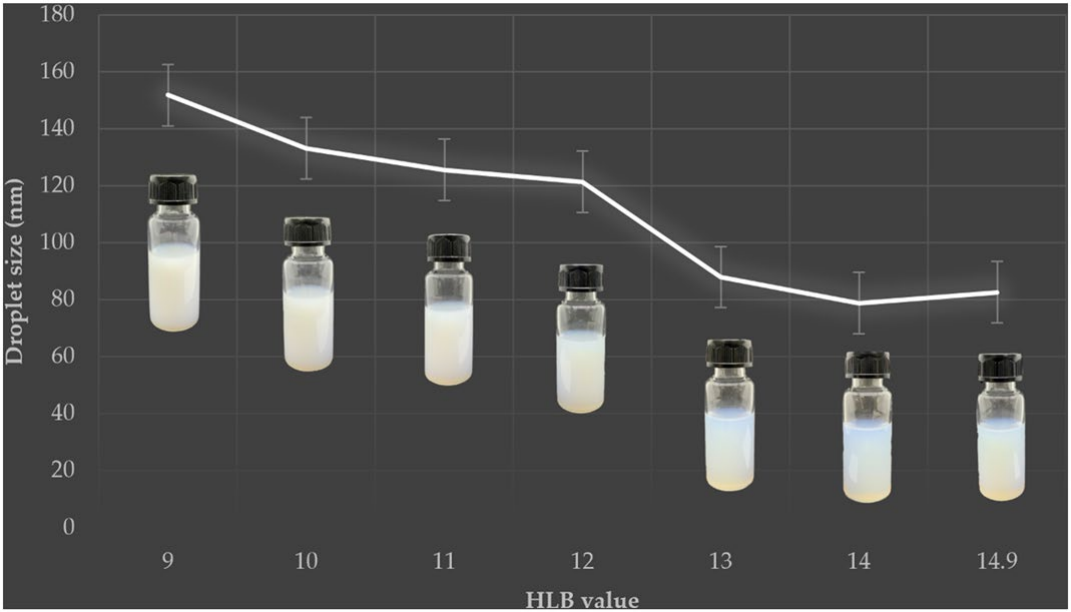
Effect of Smix HLB value on droplet size of the CEO-based nanoemulsion.

Initially, O/W nanoemulsion formulations were prepared using the high-energy technique from Smix with a range of HLB values. Ensuring an appropriate HLB value is essential for the formulation of a stable nanoemulsion. Droplet size and PI were considered when choosing the optimal formulation. A previous study has shown HLB values of 8–15 to be suitable for this type of O/W emulsion^18,29^; accordingly, this study investigated the effect of Smix with HLB values in the range of 9–14.9. DLS technique was used for the determination of the mean particle size and the particle size distribution of the nanoemulsion^1,20,30^. The nanoemulsion formulation with different particle size presented a single peak with a symmetrical distribution (data not shown). As shown in Fig. 1 and Table 3, nanoformulations with these HLB values consistently produced a mean droplet size below 200 nm, with the average droplet size decreasing as HLB value increased from 9 to 14. The smallest droplet size (79 nm) was obtained when using Smix at HLB 14. In general, increasing the Tween 60 fraction decreased droplet size; however, the formulation at HLB 14.9, which lacked a Span 60 fraction, exhibited increased droplet size (82 nm). This is consistent with prior observations that a nanoemulsion formulated with a surfactant mixture disperses and solubilizes better than one using only a single surfactant^29,31,32^.

**Table 3:** Z-average (particle diameter), PI, and zeta potential of CEO-based nanoemulsions.

HLB	Z-average (nm)	PI	Zeta potential (mV)
9	152 ± 0.6 a	0.242 ± 0.006 b	− 37.22 ± 0.33 e
10	133 ± 1.4 b	0.240 ± 0.006 b	− 23.34 ± 0.87 cd
11	126 ± 1.0 c	0.236 ± 0.007 b	− 23.16 ± 1.02 c
12	121 ± 0.8 d	0.243 ± 0.007 b	− 25.24 ± 1.29 d
13	88 ± 0.7 e	0.265 ± 0.006 a	− 16.76 ± 0.85 a
14	79 ± 0.5 g	0.276 ± 0.005 a	− 19.70 ± 1.11 b
14.9	83 ± 0.2 f.	0.277 ± 0.004 a	− 18.38 ± 1.11 ab

The optimized formulation was produced at HLB 14. Means ± standard deviations. Means with different letters within a column are significantly different (*p* < 0.05).

After preparation, the formulations made with Smix at HLB 13–14.9 appeared translucent with a blue tint and were without separated phase, flocculation, or coalescence. On the other hand, nanoemulsions at HLB 9–12 showed a separated phase. Accordingly, the optimized citronella oil-based nanoemulsion formulation was produced using Smix at HLB 14. Agrawal, et al.^29^ also formulated citronella oil into an O/W nanoemulsion by a high-energy method (ultrasonic processor) using Tween 80 and Span 80 as the surfactant mixture. They likewise obtained a minimum droplet size at HLB 14.

PI indicates the homogeneity of a nanoemulsion. The nanoemulsions produced here at HLB 9–14.9 had respective PI values of 0.261, 0.276, 0.269, 0.302, 0.286, and 0.307.

In the present study, zeta potential was not considered as a criterion for selecting the optimal nanoemulsion. Table 3 lists the zeta potential values obtained for CEO nanoemulsions with HLB 9–14.9. A zeta potential of >  + 30 or <  − 30 mV confirms that the nanoemulsion is stable, representing a high energy barrier toward the coalescence of dispersed droplets. However, this threshold is based on experiments and is not the only indicator for predicting nanoemulsion stability. The low zeta potential values in this work might be attributable to the use of nonionic surfactants (Tween 60 and Span 60)^33^. The O/W nanoemulsion consisted of CEO was coated by Tween 60 and Span 60, which should have no charge. But, it showed a negative charge (Table 3). The explanation may be the preferential adsorption of anions (-OH) from the water phase and due to associated with the polyoxyethylene group^34,35^. An alteration of the surface electrotatic double layer of nanoemulsion droplets due to some related oxygen atom in surfactant mixture and EO^33^.

Selecting a suitable Smix is very necessary as it influences droplet characteristics. As the nanoparticle formulation with the smallest diameter was produced from Tween 60 (91.2%) and Span 60 (8.8%), the rHLB value for CEO was determined as HLB 14. This formulation was selected for evaluation in further experiments.

### Characterization of the selected formulations

#### Effect of Smix concentration on nanoemulsion droplets

In the present study, Smix (HLB 14) concentration was varied over a range of 0.5–4%., while that of the dispersed phase (CEO) was held constant at 2%. The effect of Smix concentration on nanoemulsion droplet size, PI, and zeta potential value is summarized in Table 4. Increasing the proportion of Smix from 0.5 to 2% significantly reduced droplet size (from 117 to 79 nm); however, further increasing Smix to 4% increased droplet size (86 nm). Notably, the nanoemulsion prepared with a Smix concentration of 2% appeared to be translucent, but that using 4% Smix became more turbid. Carpenter and Saharan^18^ previously reported that increasing surfactant fraction resulted in an insufficiency of the interfacial sites that lead to micellization of surfactant molecules in the water phase, which generated increased turbidity of emulsion.

**Table 4:** Z-average (particle diameter), PI, and zeta potential of CEO-based nanoemulsions incorporating different concentrations of Smix at HLB 14.

Smix concentration (%)	0.5	1	2	4
Z-average (nm)	117 ± 0.5 a	89 ± 0.7 b	79 ± 0.5 d	86 ± 0.8 c
PI	0.147 ± 0.018 d	0.208 ± 0.007 c	0.276 ± 0.005 b	0.303 ± 0.004 a
Zeta potential (mV)	− 22.86 ± 0.94 d	− 9.31 ± 0.52 a	− 19.70 ± 1.11 c	− 16.53 ± 0.53 b

Means ± standard deviations. Means with different letters within a row are significantly different (*p* < 0.05).

Greater Smix concentration also influenced other characteristics such as PI and zeta potential (Table 4); for example, when increasing Smix concentration from 0.5 to 4%, the PI value also increased from 0.1472 to 0.3030.

#### Surface tension and pH value

The optimal formulation of CEO-based nanoemulsion, namely that having the smallest particle diameter, was further evaluated and characterized. It exhibited a surface tension of 31.67 mN/m, which will assist in easy delivery of the essential oil to plants as an agrochemical due to possessing higher wetting, spreading, and penetrating properties^36^. Solution pH was evaluated as a stability indicator, as most pesticide nanoemulsions degrade with an alkaline solution or a high pH value^37^. In the present study, the selected nanoemulsion had a mildly acidic pH value (pH 5.1), indicating that it could be stable.

#### Rheology

**Figure 2:**
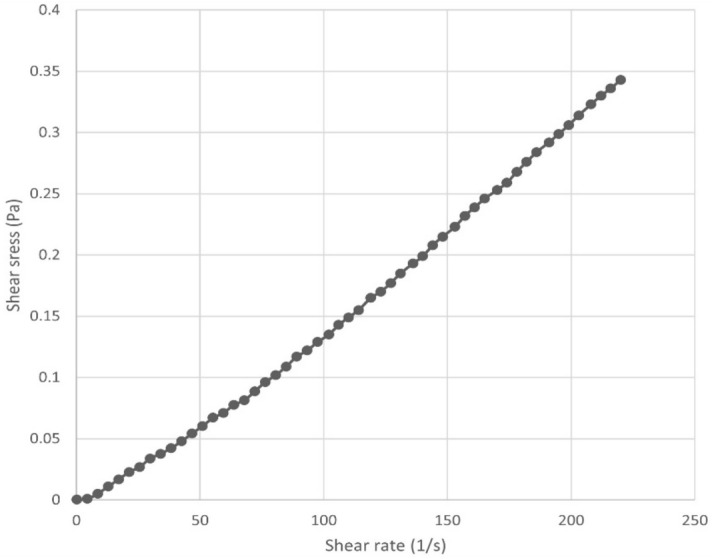
Flow curve a shear stress/shear rate of citronella nanoemulsion.

In Fig. 2, the Newtonian model was suitable model to describe the flow behavior of the citronella nanoemulsion. Similarly, Hashtjin and Abbasi^38^ reported that the rheological behavior of the orange peel nanoemulsion presented the Newtonian model with linear relationship of shear stress and shear rate.

#### Morphology

**Figure 3:**
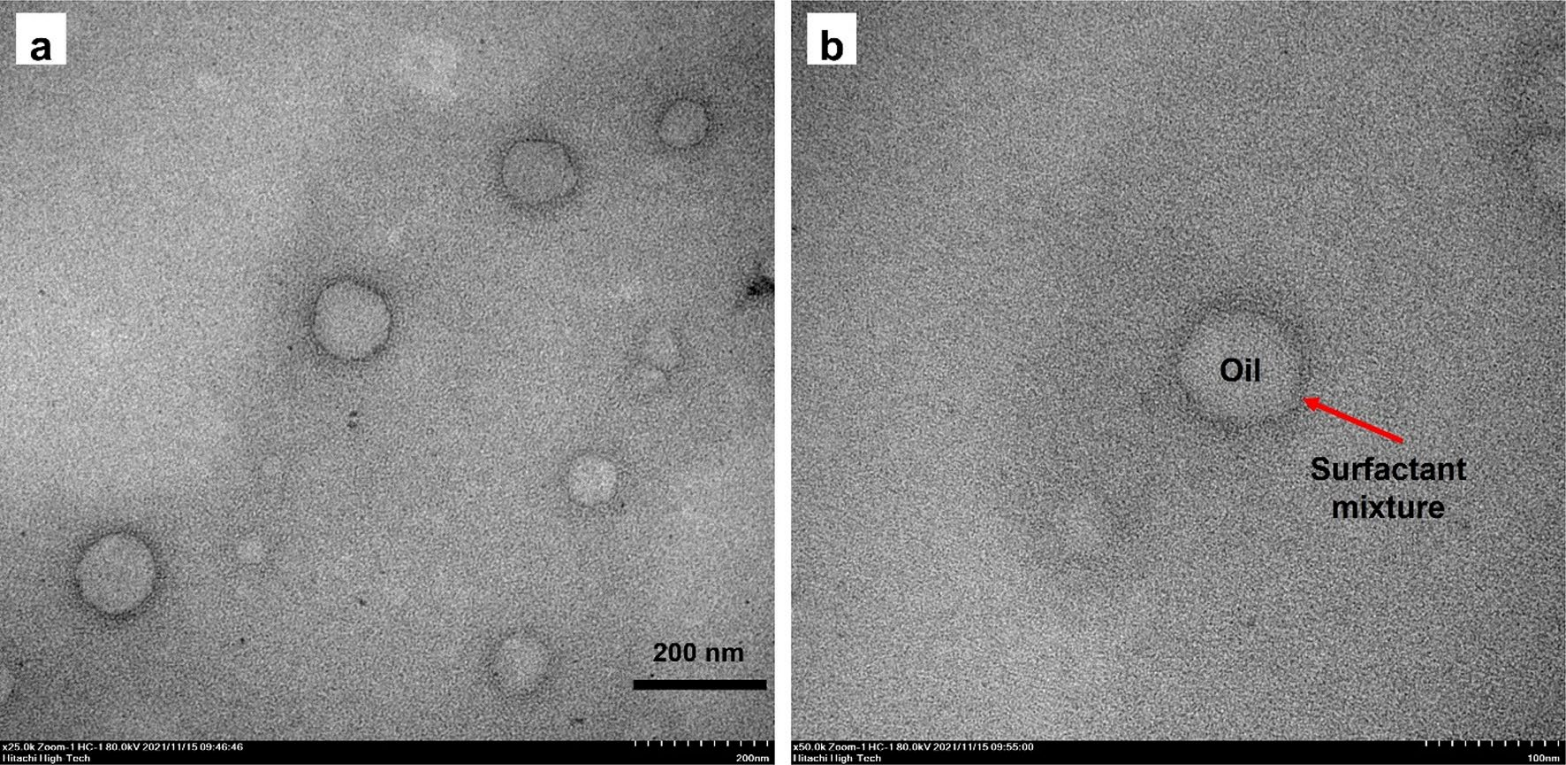
Representative TEM pictures of the optimized CEO-based nanoemulsion. (**a**) A droplet cluster. Scale bar represents 200 nm. (**b**) A single droplet. Scale bar represents 100 nm.

To confirm droplet size in the selected O/W nanoemulsion, droplet shapes were investigated through transmission electron microscopy (TEM) images, of which representative examples are shown in Fig. 3. The morphology of nanodroplet clusters in the nanoemulsion was spherical, with an interior gray part (oil) surrounded by a black ring (surfactant). In the pictures, nanodroplet size ranged 50–120 nm and correlated to the average droplet size as determined by the DLS technique. Similarly, Kumari, et al.^39^ reported TEM analysis of a thymol nanoemulsion to show spherical droplets of 80–150 nm.

#### Storage stability of the CEO nanoemulsion

Temperature influences the stability of emulsions that has an effect on the physical properties of oil, water and surfactant^40^. The optimal formulation (HLB 14) was investigated with regard to the effect of temperature and storage time on stability of the CEO nanoemulsion. The chosen formulation was stored at temperatures of 4, 25, and 45 °C, and the droplet size, PI, and zeta potential value were assayed every seven days over a total storage period of 28 days. The changes in droplet size during storage at each tested temperature are given in Table 5. At a temperature of 4 °C, the mean droplet size was good over time; however, emulsions stored at 45 °C exhibited greater droplet size, increasing from 79 to 218 nm after 14 days. Droplet formed larger droplet size during storage due to related Ostwald ripening process, which dominates the destabilization of nanoemulsions^17,41–43^. More, increasing droplet size at high temperature may be because of Brownian motion of dispersed droplets resulting in coalescence or flocculation^44^. Furthermore, separation of phases was seen after 21 days at 45 °C. Borba, et al.^41^ previously described that centrifugal force and high temperature could accelerate Brownian motion. Hence, droplets may move close to each other, causing an opportunity for destabilization with enlarged droplet size. Similarly, Falleh, et al.^45^ reported that their nanoemulsions exhibited a great droplet size with no separation phase at 6, 14, and 20 days of storage at 4 °C. Also, Masdor, et al.^46^ reported that their nanoemulsion from *Cinnamomum zeylanicum* essential oil showed stability at 4–30 °C for 60 days. However, while separation was observed after storage for 21 days at 45 °C, the nanoemulsion remained stable (< 200 nm) after just 7 days at that temperature. Meanwhile, for the nanoemulsion stored at room temperature, droplet size changed only slightly over the full 28-day storage period (Table 5). These results indicate that temperature has substantial influence on nanoemulsion droplet size and related properties, including PI. The size distribution decreased with storage time, reaching values below 0.276 in all treatments. These results agree with Teng, et al.^47^, which reported that storage temperature and time duration are the most important factors influencing nanoemulsion stability. Namely, the nanoemulsion gradually oxidizes during storage, and the degree of oxidation increases with storage time and temperature. Lipid oxidation might change the interfacial composition of the nanoemulsion, causing the emulsifier to rearrange and desorb at the interface and hence reducing the stability of the emulsion system^47,48^. Eventually, the stored nanoemulsions break up, which causes CEO to be released from the nanoemulsion. Particle size is a significant parameter in ensuring the physical stability of nanodroplet formulations under storage.

**Table 5:** The effect of storage temperature and duration on Z-average (particle diameter), PI, and zeta potential of CEO-based nanoemulsions.

Duration (days)	0	7	14	21	28
**Z-average (nm)**					
4 °C	–	60 ± 0.5 Ac	57 ± 0.2 Bc	58 ± 0.5 Bb	58 ± 0.4 Bb
25 °C	79 ± 0.5 Da	74 ± 0.7 Eb	87 ± 0.3 Cb	117 ± 1.0 Ba	140 ± 1.8 Aa
45 °C	–	106 ± 0.8 Ba	218 ± 0.8 Aa	–	–
**PI**					
4 °C	–	0.190 ± 0.006 Bb	0.197 ± 0.005 Ba	0.190 ± 0.009 Ba	0.224 ± 0.008 Aa
25 °C	0.276 ± 0.005 Aa	0.215 ± 0.004 Ba	0.172 ± 0.009 Cb	0.103 ± 0.012 Db	0.107 ± 0.014 Db
45 °C	–	0.132 ± 0.015 Ac	0.109 ± 0.016 Bc	–	–
**Zeta potential (mV)**					
4 °C	–	− 16.60 ± 1.66 Ba	− 24.46 ± 0.75 Cb	− 16.29 ± 1.12 Bb	− 12.24 ± 0.93 Aa
25 °C	− 19.70 ± 1.11 Ba	− 21.20 ± 1.83 Bb	− 19.79 ± 0.52 Ba	− 13.92 ± 1.93 Aa	− 12.75 ± 0.53 Aa
45 °C	–	− 20.32 ± 1.08 Ab	− 19.41 ± 0.68 Aa	–	–

Means ± standard deviations. Means with different uppercase letters are significantly different (*p* < 0.05) within the row. Means with different lowercase letters are significantly different (*p* < 0.05) within the column (same parameter and day for different temperatures).

### Herbicidal activity of CEO nanoemulsion

#### Seed germination and seedling growth

**Figure 4:**
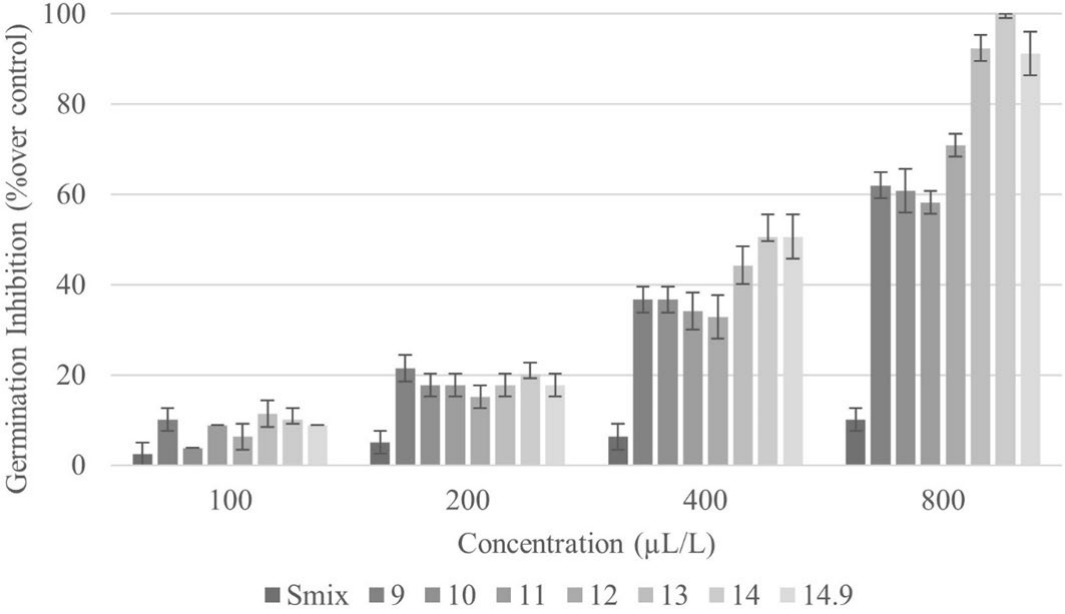
Inhibitory effect of CEO nanoemulsions with different HLB values on seed germination of *E. crus-galli*. Error bars represent standard deviation of the mean.

The effects of nanoemulsions formulated at various HLB values and concentrations on germination and seedling growth of *E. crus-galli* were investigated using the Petri dish test. Nanoemulsion HLB ranged from 9 to 14.9, and the solutions had different droplet sizes. All were found to affect germination percentage and root and shoot length in the tested weed, those parameters decreasing with increasing nanoemulsion concentration; in addition, dose-responses relationship with HLB value were observed. Seven days after treatment, the nanoemulsions at HLB 13–14.9 presented a remarkable effect on seed germination (Fig. 4). Overall, the effect of the nanoemulsion at HLB 14, which featured the smallest particle size (79 nm), was most excellent relative to solutions prepared with other HLB values. As shown in Fig. 3, treatment with HLB 14 solution at the highest tested concentration (800 µL/L of CEO) completely inhibited the germination of *E. crus-galli* seeds. However, treatment with the HLB 13 solution (droplet size 88 nm) at the same concentration also showed an inhibition of germination that was not significantly different from that obtained with HLB 14. Meanwhile, nanoemulsions at HLB 9–11 exhibited the lowest inhibitory effect on germination of *E. crus-galli* seeds, consistent with these solutions having droplet sizes above 100 nm (126–152 nm).

**Figure 5:**
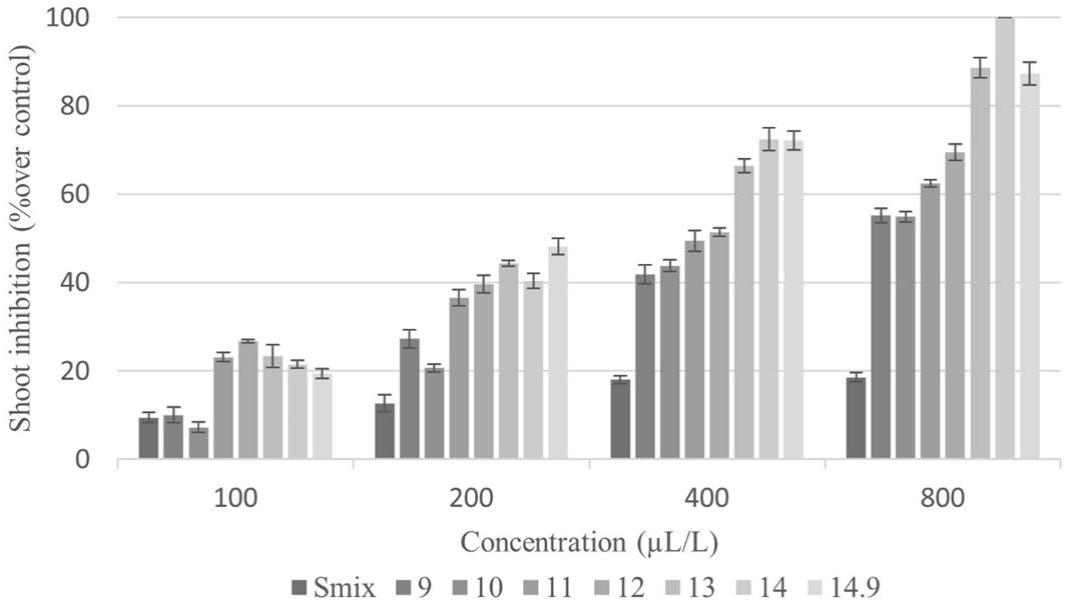
Inhibitory effect of CEO nanoemulsions with different HLB values on shoot length of *E. crus-galli*. Error bars represent standard deviation of the mean.

**Figure 6:**
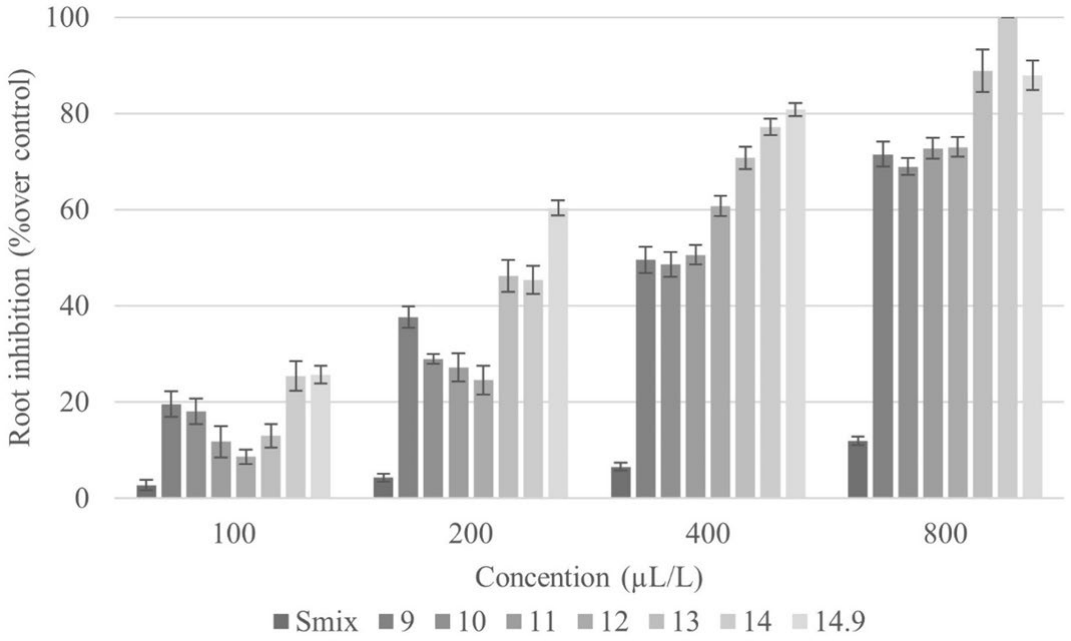
Inhibitory effect of CEO nanoemulsions with different HLB values on root length of *E. crus-galli*. Error bars represent standard deviation of the mean.

In addition to affecting seed germination, the CEO nanoemulsions also impacted seedling growth of *E. crus-galli* (Figs. 5, 6). Physical evaluation of shoot and root lengths revealed both to be reduced by nanoemulsion treatment across the range of tested HLB values. Shoot length exhibited different degrees of inhibition (Fig. 5), with the maximum shoot length being recorded in the control (data not shown). Results for root length also differed across tested HLB values and exhibited a dose-dependent response (Fig. 6). In short, CEO nanoemulsions have a dose effect on *E. crus-galli* seed germination and seedling growth, with that at HLB 14 having the highest inhibitory potential*.*

These results are in agreement with previous research indicating that nanoemulsions at similar concentrations have greater inhibitory potential on seed germination and seedling growth than ordinary emulsions (> 200 nm) due to their tiny particle size. In addition to larger particle size, other factors that constrain the allelochemical potential of oil components include evaporation and oxidation^1,36^. Accordingly, nanoemulsions have been formulated to improve the properties of essential active chemicals, ensuring their effective release and rapid interaction with plant cells after application on weed seeds or leaf surfaces^36,49,50^. In the present study, CEO was determined to consist of 95% monoterpenes, biologically active compounds that have demonstrated inhibitory potential against seed and seedling growth. Several prior reports have found monoterpenes to show various allelopathic effects on seed germination, with hydrocarbons being minor inhibitors compared to oxygenated monoterpenes^51,52^. Our results confirm that the nanoemulsion developed from CEO, Tween 60, and Span 60 by the high-energy method has enhanced potential to inhibit germination and seedling growth of *E. crus-galli*.

The inhibitory effects of this nanoemulsion on the tested seeds could be attributed to the minor and major compounds in CEO, the concentration of CEO, and the particle size of the nanoemulsion. To further study the phytotoxicity of the nanoemulsion on *E. crus-galli*, the optimal solution with HLB 14 was utilized in experimental assays.

#### Seed imbibition

**Figure 7:**
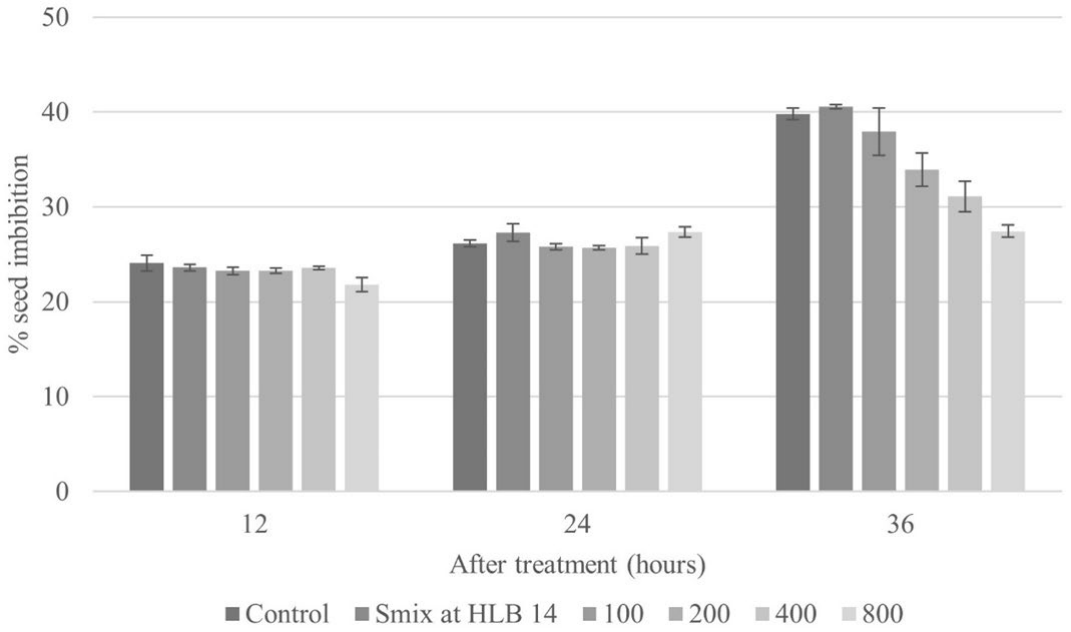
The effect of the optimized CEO nanoemulsion on imbibition of *E. crus-galli* seeds at 12, 24, and 36 h. Error bars represent standard deviation of the mean.

Seed imbibition is the initial step in the process of seed germination. The effect of the nanoemulsion formulation at HLB 14 was investigated with respect to water adsorption by the tested seeds over 12, 24, and 36 h. For a given concentration of treatment, the percentage of seed imbibition increased with imbibition period. In the first 12 h of imbibition, control seeds soaked in water showed the highest water adsorption, which was not significantly different from that obtained with Smix solution alone or most treatment solutions (100, 200, and 400 µL/L of CEO) (Fig. 7). However, treatment with the highest concentration of CEO (800 µL/L) consistently resulted in the lowest seed imbibition percentage across the time course. Moreover, at 36 h, the percentage of seed imbibition decreased with increasing concentrations of CEO.

These results are in line with observations by Teerarak, et al.^53^ from treatment of *E. crus-galli* seeds with *Aglaia odorata* Lour. essential oil, in which the inhibitory effect on imbibition was elevated for increased concentrations of oil.

#### α-Amylase activity

**Figure 8:**
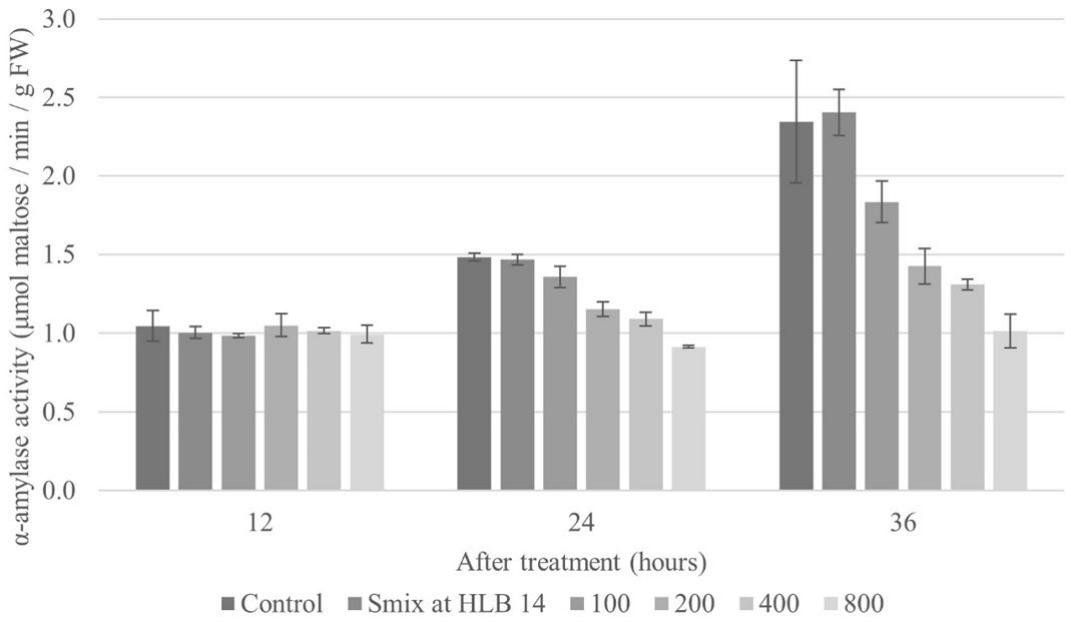
The effect of the optimized CEO nanoemulsion on α-amylase activity of *E. crus-galli* seeds at 12, 24, and 36 h. Error bars represent standard deviation of the mean.

Figure 8 presents the effect of the nanoemulsion at HLB 14 on seed germination in relation to α-amylase activity and carbohydrate degradation. In the germination process, α-amylase acts to digest starch into small organic molecules, thereby producing the nutrients and energy needed for germination^54^. To test whether a decrease in α-amylase activity mediated CEO-induced seed germination inhibition, the effect of the CEO nanoemulsion at concentrations of 100, 200, 400, and 800 µL/L on α-amylase activity was investigated. Overall, CEO-based nanoemulsions reduced α-amylase activity of the tested weed seeds relative to the water control. After 12 h, the nanoemulsion-treated samples were not significantly different from the control; however, after 24 and 36 h, α-amylase activity was significantly decreased in a dose-dependent manner. Mainly, the highest tested CEO concentration (800 µL/L) favorably inhibited α-amylase activity, which is consistent with the results of the imbibition assay (Fig. 6). Poonpaiboonpipat, et al.^55^ similarly investigated the mechanism by which *Cymbopogon citratus* EO inhibits seed germination. They reported that the α-amylase activity of *E. crus-galli* seeds treated with the oil was decreased. Similarly, Laosinwattana, et al.^56^ investigated the inhibitory effect of *Tagetes erecta* L. EO on α-amylase activity of *E. crus-galli* seeds using an emulsifiable concentrate of the EO (EC-EO). Their report indicated that the EO could inhibit α-amylase activity in a dose-dependent manner, with the highest tested concentration (2000 µL/L of EC-EO) exhibiting a significantly outstanding decrease of seed α-amylase activity at 48 h.

The results of the present study show that inhibition of α-amylase activity is one of the herbicidal activities of CEO nanoemulsions. This effect could in turn cause inhibition of seed germination and seedling growth due to starch not being degraded into small molecules to fuel growth and development.

## Conclusion

In this study, CEO was determined to be mainly comprised of monoterpenes. The formulation, stability, and some herbicidal activities of the O/W CEO-based nanoemulsion were provided and an optimum formulation was determined, along with its herbicidal efficacy against *E. cruss-galli*. It was observed that the high-pressure homogenization method with Tween 60 and Span 60 at HLB 14 produces an emulsion that has the smallest droplet size, can be stable during storage at different temperatures, and exerts the highest inhibitory effect on germination and seedling growth of *E. cruss-galli*. Regarding inhibitory effects, bioherbicidal activity assays showed the optimized CEO-based nanoemulsion to exhibit strong phytotoxic effects on seed germination, plant development, reduction of seed imbibition, and α-amylase activity.

The above-mentioned results encourage the use of CEO nanoemulsion as a natural herbicidal product for sustainable weed management. The authors expect that the present work supports exploration of bioherbicides as a green alternative to solve the problems of pollution in the environment and human health.
